# Dr. Chester A. Meyer

**Published:** 1915-05

**Authors:** 


### OBITUARY

Readers are requested to report promptly the death of all physicians in Western New York, or former residents of this region, or graduates of any medical school in Western New York, and to notify the families of the deceased of our desire to publish adequate obituary notices.

**Dr. Chester A. Meyer,** N. Y. Homoeopathic, 1881, died at his home in Louisville, April 10. He was born in Buffalo, Jan. 27, 1857, graduated at the Buffalo Central School and was appointed, on competitive examination, to the U. S. Naval Academy at Annapolis, resigning to take up the study of medicine. He had held many positions of honor.

